# Quality of Antimicrobial Products Used in Striped Catfish (*Pangasianodon hypophthalmus*) Aquaculture in Vietnam

**DOI:** 10.1371/journal.pone.0124267

**Published:** 2015-04-21

**Authors:** Tran Minh Phu, Nguyen Thanh Phuong, Marie-Louise Scippo, Anders Dalsgaard

**Affiliations:** 1 Department of Aquatic Nutrition and Products Processing, College of Aquaculture and Fisheries, Can Tho University, Can Tho, Vietnam; 2 Department of Veterinary Disease Microbiology, Faculty of Health and Medical Sciences, University of Copenhagen, Copenhagen, Denmark; 3 Department of Food Sciences, Faculty of Veterinary Medicine, University of Liège, Liège, Belgium; University of Aveiro, PORTUGAL

## Abstract

Antimicrobial usage is common in Asian aquaculture. This study aimed to determine the quality of antimicrobial products used by Vietnamese striped catfish (*Pangasianodon hypophthalmus*) farmers. Twenty one antimicrobial products (11 products contained a single antimicrobial and 10 products contained a mixture of two different antimicrobials) commonly used by catfish farmers were obtained from so-called chemical shops located in the Mekong Delta, Vietnam. Ultra High Performance Liquid Chromatography Mass Spectrometry was used to analyze concentration of sulfonamides, trimethoprim, amoxicillin, cefalexin and ciprofloxacin whereas concentrations of florfenicol and doxycycline were analyzed by High Performance Liquid Chromatography with UV detection. Results revealed that only 4/11 products with a single antimicrobial and 2/10 products with a mixture of antimicrobials contained active substances within ±10% of the concentration declared on the product label. Two products with antimicrobial mixtures did not contain any of the declared antimicrobials. Comparing two batches, analysis of 11 products revealed that only one product contained a concentration of active compound that varied with less than 10% in both batches. Several product labels provided inadequate information on how to calculate therapeutic dosage and further stated withdrawal time despite lack of pharmacokinetic data on the antimicrobials in catfish. There is an urgent need to strengthen approval procedures and in particular regularly to monitor the quality of antimicrobials used in Vietnamese aquaculture.

## Introduction

Striped catfish (*Pangasianodon hypophthalmus*) aquaculture in Mekong Delta, Vietnam produced 1.2 million tons in 2013, but disease outbreaks, mainly Bacillary Necrosis of Pangasius (BNP) and Motile Aeromonad Septicaemia (MAS), causes mortalities up to 60% [1, 2]. Farmers prepare their own medicated feed to treat these bacterial diseases as commercial medicated feed is not available and vaccines not widely used [2]. In 2011, it was estimated that 106 tons of antimicrobials were used in striped catfish aquaculture representing 20 antimicrobials, mainly β-lactams, phenicols, quinolones and sulfonamides [2, 3]. Inferior quality of antimicrobial products may lead farmers to provide medications at sub-therapeutic dosage and subsequent to treatment failure and development of antimicrobial resistance [4]. In contrast to antimicrobial products used for treatment of humans, little documentation is available about the quality of antimicrobial products used in aquaculture. This study therefore aimed to determine the quality of common antimicrobial products used by Vietnamese striped catfish farmers.

## Materials and Methods

### Collection of antimicrobial products

Twenty one antimicrobial products marketed by ten Vietnamese companies were obtained during visits to nine so-called chemical shops located in three major catfish producing provinces in the Mekong Delta, Vietnam. All products were produced in Vietnam. Information about the products is presented in Table 1. Two different batches of 11 products were obtained for comparison analysis. The selected products represented the most commonly used antimicrobials by the catfish farmers and included 11 products with a single antimicrobial (amoxicillin, doxycycline or florfenicol) and 10 products with a mixture of two antimicrobials (Table 1). Seventeen antimicrobial products were in powder form and the remaining in liquid form. All products had an expiry date up to two years and antimicrobial content analysis was done at least one year before expiry date. Samples were stored at ambient temperature, e.g. like in the chemical shops, until laboratory analysis.

**Table 1:** Information about the 21 antimicrobial products analyzed.

Company code	Antimicrobial type and concentration declared on product label ^a^	Weight—volume	Form	Type of package	Sampling location (district, province)
I	SMX 20% + TMP 4%	500 g	Powder	Aluminium bag	Cao Lanh, Dong Thap
II	SMX 20% + TMP 4%	100 g	Powder	Aluminium bag	Cao Lanh, Dong Thap
III	SMX 20% + TMP 4%	100 g	Powder	Aluminium bag	Ninh Kieu, Can Tho
V	SMX 20% + TMP 4%	1000 g	Powder	Aluminium bag	Cai Rang, Can Tho
IV	SDZ 33% + TMP 6.7%	1000 g	Powder	Aluminium bag	Thot Not, Can Tho
III	DOX 5% + FFC 10%	250 mL	Liquid	Glass bottle	Ninh Kieu, Can Tho
IV	DOX 10% + FFC 20%	1000 mL	Liquid	Plastic bottle	Thot Not, Can Tho
VI	FFC 40% + LEX 35%	200 g	Powder	Aluminium bag	Long Xuyen, An Giang
VI	FFC 20% + AMX 5%	100 g	Powder	Aluminium bag	Long Xuyen, An Giang
VII	AMX 12% + CIP 10%	1000 g	Powder	Aluminium bag	Thot Not, Can Tho
I	FFC 10%	100 g	Powder	Aluminium bag	Cao Lanh, Dong Thap
II	FFC 5%	100 g	Powder	Aluminium bag	Cao Lanh, Dong Thap
VIII	FFC 5%	1000 g	Powder	Aluminium bag	Thot Not, Can Tho
III	FFC 5%	250 mL	Liquid	Glass bottle	Ninh Kieu, Can Tho
III	FFC 20%	250 mL	Liquid	Glass bottle	Ninh Kieu, Can Tho
II	DOX 5%	100 g	Powder	Aluminium bag	Cao Lanh, Dong Thap
IX	DOX 10%	100 g	Powder	Aluminium bag	Cao Lanh, Dong Thap
V	DOX 10%	1000 g	Powder	Aluminium bag	Cai Rang, Can Tho
III	DOX 20%	1000 g	Powder	Plastic bottle	Ninh Kieu, Can Tho
III	AMX 10%	100 g	Powder	Aluminium bag	Ninh Kieu, Can Tho
X	AMX 15%	100 g	Powder	Aluminium bag	Ninh Kieu, Can Tho

^a^ SMX: sulfamethoxazole; SDZ: sulfadiazine; TMP: trimethoprim; DOX: doxycycline; FFC: florfenicol; AMX: amoxicillin; LEX: cefalexin; CIP: ciprofloxacin.

### Antimicrobial analysis

Coded samples without product labels were analyzed at the National Agro-Forestry Fisheries Quality Assurance Department (NAFIQAD) laboratory in Can Tho, Vietnam which is ISO/IEC 17025 accredited [5]. Solutions of antimicrobial products were analyzed using chromatographic separation conditions and detection parameters as described previously [6–10]. Ultra High Performance Liquid Chromatography Mass Spectrometry (UPLC-MS/MS) was used for sulfamethoxazole (SMX); sulfadiazine (SDZ); sulfamethazine (SMZ) and trimethoprim (TMP) [6]; amoxicillin (AMX) and cefalexin (LEX) [7]; and ciprofloxacin (CIP) [8] whereas concentrations of florfenicol (FFC) [9] and doxycycline (DOX) [10] products were analyzed by High Performance Liquid Chromatography with UV detection (HPLC-UV). Each sample was analyzed in duplicate. The variation between compound concentrations of duplicate sample analysis was less than 5%. The matrix effect observed showed that there was no matrix effect when the antimicrobial products were analyzed. Analytical results were presented as the percentage (%) of active antimicrobial compound concentration as compared with the concentration declared on product label.

#### Materials and reagents

Standards (purity >98%) of sulfadiazine, sulfamethazine, sulfamethoxazole, sulfapyridine, sulfamerazine, sulfachloropyridazine, sulfadimethoxine, sulfadoxine, sulfachinoxaline, sulfathiazole, sulfamethizole, trimethoprim, florfenicol, doxycycline, amoxicillin, cefalexin, penicillin V, sarafloxacin d8 and ciprofloxacin were purchased (Dr. Ehrenstorfer, Augsburg, Germany) and stored at 4°C. HPLC water, acetonitrile, formic acid 98%, ammonium formate (Merck, Darmstadt, Germany) and acetic acid 100% were obtained from J.T.Baker, Mallinckrodt Inc, MO, USA. All antimicrobial stock solutions (1000 μg/mL) were prepared in methanol excepted AMX and LEX (1000 μg/mL) which were prepared in methanol and water (1:1). Stock solutions were then diluted up to 1 μg/mL for standard curve preparation.

#### Sample preparation

Each antimicrobial product was prepared by diluting 1 g in 100 mL appropriate solvent, which was then diluted by adding HPLC mobile phase solution specific for each class of antimicrobial. For analysis of sulfonamides and TMP, samples were spiked with sulfapyridine as internal standard (IS), at a concentration of 100 ng/mL. Penicillin V (200 ng/mL spiked concentration) was used as IS for analysis of AMX and LEX. Sarafloxacin-d8 (100 ng/mL) was used as IS for CIP analysis. Samples were filtered through a 0.22 μm nylon membrane filter (Advantec MFS, CA, USA). The filtrate was then injected into the HPLC-UV system (Water Alliance 2690, USA) for FFC and DOX analysis and into a UPLC-MS/MS system (Waters ACQUITY, Milford, MA, USA) for analysis of the other antimicrobials.

#### Determination of sulfonamides, trimethoprim, ciprofloxacin, cefalexin and amoxicillin by UPLC-MS/MS

The Waters ACQUITY UPLC system (Waters, Milford, MA, USA) was used for sulfonamides, trimethoprim (TMP), ciprofloxacin (CIP), cefalexin (LEX) and amoxicillin (AMX) analysis. For sulfonamides, TMP and CIP, separation of compounds was performed on a 3×100 mm column packed with 1.7 μm particles (ACQUITY UPLC BEH C18 column, Waters, USA) in combination with the same guard column (Waters, USA), both maintained at 40°C. For sulfonamides and TMP, a gradient program was established to control the mobile phases A (100% acetonitrile) and B (1%, v: v, formic acid in water). Initial gradient conditions were set to 20% A and held for 1 min before incorporating a linear gradient increasing to 60% A at 4 min. At 4.2 min, the gradient was programmed to initial conditions to re-equilibrate for 3 min before a new sample injection. The flow rate was 0.3 mL/min and the injection volume was 10 μL in full loop injection mode. For analysis of CIP, a gradient program was established to control the mobile phases A (acetonitrile) and B (2mM ammonium formate, pH 3). Initial gradient condition was set to 10% A and hold for 1 min before increasing to 30% A at 2 min, 40% A at 2.5 min and 60% A at 3 min. At 3.2 min, the gradient was programmed to initial conditions to re-equilibrate for 2 min before a new sample injection. The flow rate was 0.25 mL/min and the injection volume was 10 μL in full loop injection mode.

For analysis of LEX and AMX, the column was an ACQUITY UPLC BEH C18 (1.7 μm, 2.1mm x 50 mm, Waters, MA, USA). Column temperature was maintained at 40°C. A gradient program was established to control the mobile phases A (acetonitrile: methalnol, 1: 1 and 0.1% formic acid) and B (0.1%, v: v, formic acid in water). Initial gradient conditions were set to 10% A and held for 1 min before incorporating a linear gradient increasing to 85% A at 1.5 min and hold it for 1.5 min. At 3.2 min, the gradient was programmed to initial conditions to re-equilibrate for 3 min before a new sample injection. The flow rate was 0.3 mL/min and the injection volume was 10 μL in full loop injection mode.

Detection and quantification were done by a Xevo TQ triple quadrupole mass spectrometry combined with electrospray ionization (ESI) probe operated in the positive ion mode (Waters, MA, USA). The mass spectrometry parameters were optimal for all mentioned compounds including capillary voltage, ion source temperature, and desolvation gas flow rate (data not shown). Detection of the compounds was carried out in multiple reactions monitoring mode. Argon was used as the collision gas. Each compound was detected following two specific fragmentations per compound. Compound mass, fragments and collision energy was optimized (data not shown). The MassLynx 4.1 (TargetLynx quantification) software was used to control the instruments as well as for data acquisition and processing for automatic quantification.

The concentrations of antimicrobial were determined using calibration curves made with solutions at six concentration levels, spiked with their respective IS (see above). All calibration curves were prepared on the same days samples were analyzed.

#### Determination of florfenicol and doxycycline by HPLC-UV

Analysis of FFC and DOX was done using an HPLC system (Water Alliance 2690, USA) connected to a UV detector set at 224 nm for FFC and 375 nm for DOX. Compound separation was done using Symmetry C18 column 5μm, 4.6 mm x150 mm (Waters, MA, USA), kept at 40°C in isocratic mode using a flow rate of 1mL/min. Six points calibration curves were used, without internal standard. Chemstation B.04.02 software was used to control the instruments as well as for data acquisition and processing for automatic quantification.

### Product label information

The information provided on products labels were evaluated with regards to information about prophylactic use, diseases to be treated, therapeutic dosage, means of calculating the dose, how to prepare medicated feed and withdrawal time.

## Results and Discussion

A total of 4/11 products with a single antimicrobial declared (company I, FFC 10%; company II, FFC 5%; company III, FFC 5% and DOX 20%) and 2/10 products containing a mixture of two declared antimicrobials (companies II and V, SMX 20% + TMP 4%) contained active substances within ±10% of the concentration declared on the product label which is the level of variation accepted by the Vietnamese authorities (Table 2) [11]. Products from company VI (FFC 20% + AMX 5%) and company VII (AMX 12% + CIP 10%) did not contain any of the declared antimicrobials. Products from company IV (SDZ 33% + TMP 6.7%) and company VI (FFC 40% + LEX 35%) contained only one of the declared active substances at less than 50% of the declared concentrations (Table 2). Further analysis of a product from company IV (SDZ 33% + TMP 6.7%) revealed SMZ instead of SDZ and AMX instead of LEX in a product from company VI (FFC 40% + LEX 35%). Furthermore, the product (FFC 20% + AMX 5%) from company VI contained LEX instead of AMX. Eight of 21 products contained between 3.00–60.7% lower antimicrobial concentrations than declared. The product from company III contained 284% of declared FFC concentration (Table 2). Comparing two batches, analysis of eleven products revealed that only one product (FFC 5%) from company III contained concentrations of the active compound that varied with less than 10% between batches (Table 2) [11].

**Table 2:** Active antimicrobial compound concentration as compared with the concentration declared on product labels (%) and therapeutic dose declared.

Company code	Antimicrobial type and concentration declared on product label ^a^	SMX	SDZ	TMP	DOX	FFC	AMX	LEX	CIP	Therapeutic dose (mg active compound/ kg fish)	Therapeutic dose (mg active compound/ kg feed)
I	SMX 20% + TMP 4%	117–104^b^	-	118–28.3	-	-	-	-	-	14.3–2.90	250–50.0
II	SMX 20% + TMP 4%	116–109	-	108–106	-	-	-	-	-	16.7–3.33	286–57.1
III	SMX 20% + TMP 4%	133–114	-	124–101	-	-	-	-	-	50.0–10.0	1000–200
V	SMX 20% + TMP 4%	96.7	-	95.8	-	-	-	-	-	-	1000–200
IV	SDZ 33% + TMP 6.7%	-	ND-ND^c^	47.7–32.4	-	-	-	-	-	10.0–2.00	-
III	DOX 5% + FFC 10%	-	-	-	24.8	284	-	-	-	2.50–5.00	50.0–100
IV	DOX 10% + FFC 20%	-	-	-	40.3	60.7	-	-	-	6.67–13.3	200–400
VI	FFC 40% + LEX 35%	-	-	-	-	45.5	-	ND^d^	-	-	1000–875
VI	FFC 20% + AMX 5%	-	-	-	-	ND	ND^e^	-	-	10.0–2.50	-
VII	AMX 12% + CIP 10%	-	-	-	-	-	ND-ND	-	ND-ND	12.0–10.0	-
I	FFC 10%	-	-	-	-	91.5–38.1	-	-	-	5.00	200
II	FFC 5%	-	-	-	-	92.8–79.2	-	-	-	10.0	167
VIII	FFC 5%	-	-	-	-	89.2–86.0	-	-	-	10.0	250
III	FFC 5%	-	-	-	-	104–105	-	-	-	10.0	200
III	FFC 20%	-	-	-	-	116	-	-	-	10.0	200
II	DOX 5%	-	-	-	49.0–54.8	-	-	-	-	5.00	75.0
IX	DOX 10%	-	-	-	56.8	-	-	-	-	-	400
V	DOX 10%	-	-	-	3.00	-	-	-	-	25.0	500
III	DOX 20%	-	-	-	92.5	-	-	-	-	40.0	667
III	AMX 10%	-	-	-	-	-	134–151	-	-	50.0	-
X	AMX 15%	-	-	-	-	-	114	-	-	30.0	-

^a^ SMX: sulfamethoxazole; SDZ: sulfadiazine; TMP: trimethoprim; DOX: doxycycline; FFC: florfenicol; AMX: amoxicillin; LEX: cefalexin; CIP: ciprofloxacin

^b^The two percentage figures represent content found in two different batches

^c^ Declared compounds not detected (ND), instead of SDZ, product contained 11.0% and 10.5% sulfamethazine

^d^ contained 10.1% AMX instead of LEX

^e^ contained 11.3% LEX instead of AMX;-, not analyzed.

Compared to human antimicrobial products in particular those marketed in developing countries [12–14], there seems to be few if any studies on quality of antimicrobial products used in aquaculture. Inferior quality of antimicrobial products will lead farmers to provide medications at sub-therapeutic dosage and subsequent to treatment failure, excess fish mortality and economic loses. Further, sub-therapeutic doses of antimicrobials is the most important factor selecting for antimicrobial resistance both among bacterial pathogens associated with specific diseases and the normal bacterial microflora, e.g. in aquaculture environments [15, 16]. Studies conducted between 2008 and 2012, showed increasing levels of antimicrobial resistance among *Edwardsiella ictaluri* and *Aeromonas hydrophila* isolated from striped catfish in Mekong Delta with both pathogens now being resistant to nearly all approved antimicrobials for use in Vietnamese aquaculture [4, 17]. Several product labels stated that the antimicrobials could be used for prophylactic treatment which will further add to the selection of antimicrobial resistance.

All labels stated that the product should be used to treat bacterial diseases while five products were also marketed for prophylactic use. The calculation of therapeutic dosage was declared to be based on fish biomass (5/21 products), amount of feed used (3/21 products) or both means of calculation (13/21 products). Correct dosage should be calculated based on the antimicrobial concentration in the prepared medicated feed together with the amount of feed consumed by a particular fish biomass. Recommended doses on labels varies highly between products, e.g. for doxycycline the dose calculated based on fish biomass was 2.50 to 6.67 and 5 to 40 mg/kg fish (in mixture and single antimicrobial products, respectively), while when calculated based on amount of feed used, the dose ranges from 50 to 200 and 75 to 667 mg/kg feed (in mixture and single antimicrobial products, respectively) (Table 2). Only the dose for florfenicol to treat *E*. *ictaluri* infections (5 and 10 mg/kg body weight declared in single antimicrobial products and 5, 10 and 13 mg/kg body weight declared in mixture antimicrobial products) was in agreement with recommended dosage for treatment of channel catfish [18, 19]. As manufactured medicated feed is not available, farmers prepare their own medicated feed which will further add to variations in concentrations of antimicrobials [20]. All product labels described how to dissolve the antimicrobial in water and subsequent mixing of antimicrobial solution with pelleted feed. It should however, be noted that some of the tested antimicrobials are only slightly soluble in water, e.g. trimethoprim and sulfamethoxazole. Labels on 18/21 products provided information about withdrawal time (15 to 30 days) despite that limited information is available about the pharmacokinetics of different antimicrobials used in striped catfish culture and consequently correct therapeutic dosage and withdrawal time have not been established for most approved antimicrobials, e.g. florfenicol, sulfonamides and tetracyclines.

All antimicrobial products analyzed were registered and approved by the Vietnamese authorities [21]. The total number of registered products for use in Vietnamese aquaculture was 2913 in 2012, including 813 so-called veterinary drugs [3]. Clearly the approval of such a high number of products demand many resources and is costly. We do not know if the inferior product quality documented may be due to inadequate testing during product approval or maybe more likely if antimicrobial composition and concentration may have been changed after product approval.

## Conclusions

In conclusion, our study documents an urgent need to strengthen approval procedures and in particular regularly to monitor the quality of antimicrobials used in Vietnamese aquaculture. The implementation of international aquaculture certification schemes which demands recording of antimicrobials used at a farm may found the basis for a national database on antimicrobial use in Vietnamese catfish culture.
